# Long-term efficacy and safety of solifenacin, mirabegron, and their combination for overactive bladder: a systematic review

**DOI:** 10.3389/fphar.2026.1874436

**Published:** 2026-06-19

**Authors:** Linlin Yang, Hongqiao Zhao, Shuna Xiao, Ziqi Ye

**Affiliations:** 1 Department of Pharmacy, The First Affiliated Hospital, and College of Clinical Medicine of Henan University of Science and Technology, Luoyang, China; 2 Department of Clinical Pharmacy, The First Affiliated Hospital, Zhejiang University School of Medicine, Hangzhou, China

**Keywords:** meta-analysis, mirabegron, overactive bladder, solifenacin, systematic review

## Abstract

**Background:**

Current clinical guidelines recommend either muscarinic receptor antagonists or β3-adrenoceptor agonists for overactive bladder (OAB) treatment. These recommendations, however, are primarily based on short-term (12-week) clinical trials, leaving a critical gap in evidence regarding their long-term (≥1 year) efficacy and safety. Moreover, the long-term evidence synthesized here is limited to solifenacin and mirabegron, and its generalizability to other agents remains uncertain.

**Objective:**

To systematically compare the long-term efficacy and safety of solifenacin monotherapy, mirabegron monotherapy, and their combination in patients with OAB.

**Methods:**

We systematically searched PubMed, Embase, Cochrane Library, Scopus, and ClinicalTrials.gov for clinical studies published from inception to 2 April 2026. Two reviewers independently screened, selected, and extracted data. Methodological quality was assessed using the Jadad scale and the Cochrane Risk of Bias 2 (ROB 2) tool. The primary outcomes were changes from baseline in mean volume voided incontinence episodes per 24 h, and micturition frequency per 24 h. The secondary outcome was the incidence of treatment-related adverse drug reactions Quantitative meta-analysis was performed only for safety outcomes using a random-effects model. For the primary efficacy outcomes, substantial heterogeneity (I^2^>58%) across the included studies precluded meaningful pooling, therefore, a narrative synthesis of efficacy data was presented.

**Results:**

Initial screening identified 7,451 articles, of which five randomized controlled trials met the inclusion criteria. For long-term OAB management, combination therapy (solifenacin 5 mg + mirabegron 50 mg) may be more effective than each monotherapy in improving MVV and reducing mean incontinence episodes and micturition frequency. Compared to mirabegron 50mg, solifenacin 5 mg was associated with a potentially higher risk of ADRs such as dry mouth (RR 1.73, 95%CI 0.91–3.30, P = 0.10) and constipation (RR 2.46, 95%CI 1.16–5.19, P = 0.02), but a lower incidence of dizziness (RR 0.08, 95%CI 0.01–0.62, P = 0.02). Solifenacin 5 mg was associated with a lower risk of constipation and dry mouth compared to solifenacin 10 mg.

**Conclusion:**

For long-term OAB pharmacotherapy with the specific regimens evaluated, mirabegron 50 mg appears to offer a favorable profile due to its comparable efficacy and lower incidence of certain treatment-limiting ADRs. However, these findings cannot be generalized to other muscarinic receptor antagonists or β3-adrenoceptor agonists.

**Systematic Review Registration:**

https://www.crd.york.ac.uk/PROSPERO/view/CRD420251056532, identifier CRD420251056532.

## 1 Introduction

Overactive bladder (OAB) is a clinical syndrome defined by urinary urgency (Beder et al., 2021), often accompanied by increased daytime frequency and nocturia, with or without urgency incontinence, in the absence of urinary tract infection or other obvious pathology (Li et al., 2022). Its prevalence ranges from 9.6% to 35.6% (Eapen and Radomski, 2016), and poor symptom control can significantly impair quality of life. While the primary goal of OAB treatment is symptom alleviation, most clinical trials have focused on short-term efficacy (12 weeks), leaving a gap in evidence regarding long-term (≥1 year) safety and efficacy.

First-line pharmacotherapies include muscarinic receptor antagonists (e.g., solifenacin, tolterodine) and β3-adrenoceptor agonists (e.g., mirabegron, vibegron) (Bowman et al., 2024). Muscarinic receptor antagonists inhibit detrusor overactivity by blocking acetylcholine binding, while β3-adrenoceptor agonists promote bladder relaxation and storage *via* β3-adrenergic receptor activation. Both drug classes are recommended for short-term OAB management. Combination therapy may be considered if monotherapy is insufficient, though it carries a potential for increased adverse drug reactions (ADRs) (Park et al., 2024). Previous research has confirmed no pharmacokinetic interaction between these classes (Nomura et al., 2016), and a 12-week study has shown combination therapy to be more efficacious with a comparable safety profile to monotherapy (Abrams et al., 2015).

The AUA/SUFU guidelines do not specify a preferred first-line pharmacotherapy, potentially leading to clinical equipoise and varied prescribing practices. The choice between initiating treatment with a muscarinic receptor antagonist or a β3-adrenoceptor agonist may lead to different clinical outcomes, creating uncertainty for physicians. Furthermore, optimal OAB management often requires sustained pharmacological intervention. This long-term strategy necessitates a careful balance between efficacy and safety to prevent treatment discontinuation due to inadequate response or intolerable ADRs (He et al., 2023; Mostafaei et al., 2022; Luo et al., 2012). Significant heterogeneity in short-term (12-week) outcomes underscores the need for extended-duration (≥1 year) evaluations. However, the current evidence base for long-term treatment is limited, preventing comprehensive comparisons across all available options (Gratzke et al., 2018; Mueller et al., 2019; Ozkidik et al., 2019; Haab et al., 2005; Staskin and Te, 2006). Notably, the long-term evidence base is currently concentrated on solifenacin and mirabegron, comparable data for other agents in these classes are lacking. Therefore, this review focuses specifically on these two agents and their combination and does not address other first-line OAB drugs.

Recently, mirabegron has been investigated beyond its approved OAB indication. For example, a systematic review and meta-analysis evaluated its efficacy and safety in patients with heart failure, reflecting growing interest in its cardiovascular applications (Abdelhamid et al., 2026). Furthermore, a recent review focused on frail older adults concluded that mirabegron remains an acceptable alternative to antimuscarinic treatment in this vulnerable population (Shaw and Wagg, 2026). In parallel, real-world data from a non-interventional study of a generic prolonged-release mirabegron formulation confirmed its favorable long-term tolerability and compliance profile in adults with OAB (Lee et al., 2026). Taken together, these emerging data underscore the continued relevance of mirabegron in both urological and non-urological fields and reinforce the need for a comprehensive long-term evaluation of its efficacy and safety relative to solifenacin and their combination.

Given the relatively abundant long-term data comparing solifenacin 5 mg with solifenacin 10mg, mirabegron 50mg, and their combination, this study focuses on a comprehensive evaluation of these regimens. By comparing their long-term efficacy and safety, we aim to provide supplementary evidence to inform initial treatment decisions for OAB. Specifically, this systematic review aimed to: (1) narratively synthesis the long-term efficacy of solifenacin 5mg, mirabegron 50mg, and their combination; and (2) quantitatively compare their safety profiles *via* meta-analysis of ADRs.

## 2 Materials and methods

### 2.1 Study design

This systematic review and meta-analysis is reported in accordance with the Preferred Reporting Items for Systematic Reviews and Meta-Analyses (PRISMA) 2020 guidelines (Page et al., 2021). As this study involved the synthesis of data from previously published and ethically approved trials, no further ethical approval or patient consent was required. The review protocol was registered with PROSPERO (Registration number: CRD420251056532).

### 2.2 Eligibility criteria

Study selection was guided by the PICOS framework as detailed below.

Population: Studies enrolling adults (≥18 years) diagnosed with OAB were included. No restrictions were applied regarding gender, OAB subtype (wet or dry), or baseline symptom severity.

Intervention: Administration of solifenacin 5 mg for a duration of at least 1 year.

Comparators: Eligible comparators included standard care, no intervention, placebo, or other active pharmacological treatments. For the purposes of this review, studies were grouped for synthesis based on the following comparator regimens: (1) solifenacin 10mg; (2) mirabegron 50mg; and (3) combination therapy (solifenacin 5 mg + mirabegron 50 mg).

Outcomes: The primary outcomes were changes from baseline to end of treatment (EOT) in: (1) mean volume voided (MVV) per micturition; (2) mean number of incontinence episodes per 24 h; and (3) mean number of micturition events per 24 h. The secondary outcome was the incidence of treatment-related ADRs.

### 2.3 Search strategy

We systematically searched the following databases and registers from inception to 2 April 2026: PubMed, Embase, Cochrane Library, Scopus, and ClinicalTrials.gov. In addition, we manually screened the reference lists of all included studies and relevant review articles to identify any additional eligible studies. The search strategy combined keywords including ‘solifenacin’, ‘overactive bladder’, ‘OAB’, and ‘long-term’. The full search strategy for each database, including all filters and limits used, is available in Supplementary Table S1. No language or publication date restrictions were applied during the search.

### 2.4 Study selection

Two reviewers (LY and HZ) independently screened titles and abstracts against the eligibility criteria. Duplicate records were removed using Endnote. Non-randomized studies, case reports, reviews, editorials, conference abstracts, and studies without extractable outcome data were excluded. Full texts of potentially eligible articles were then retrieved and independently assessed by the same two reviewers. Any disagreements were resolved through discussion or consultation with a third reviewer (ZY). If eligibility could not be determined due to missing information, we attempted to contact the study authors for clarification.

### 2.5 Data extraction

Two reviewers (LY and HZ) independently extracted data from the included studies using a standardized data extraction form. Any disagreements were resolved by consensus or by a third reviewer (ZY). If data were missing or unclear, we attempted to contact the corresponding authors of the original studies to obtain the required information.

Data items:

Outcomes: For all included studies, we sought data on the following outcomes measured from baseline to end of treatment (≥1 year): (1) change in MVV per micturition; (2) change in mean number of incontinence episodes per 24 h; (3) change in mean number of micturition events per 24 h; and (4) incidence of treatment-related ADRs. For efficacy outcomes, we sought results for all time points reported at or beyond 52 weeks. If multiple time points were available, we used the longest follow-up duration.

Other variables: We extracted the following additional information from each included study: first author, publication year, country, study design, sample size, patient characteristics (age, gender), intervention and comparator details (dose, frequency, duration), funding source, and any conflicts of interest declared by the study authors. For any missing or unclear information, we assumed that the study did not collect or report that information.

### 2.6 Assessment of risk of bias in included studies

Two reviewers (LY and HZ) independently assessed the methodological quality of the included RCTs using the Jadad scale (McCormick et al., 2013). This scale evaluates three domains: randomization (description and appropriateness), double-blinding (description and appropriateness), and reporting of withdrawals and dropouts. Each domain is scored from 0 to 2, with total scores ranging from 0 to 5. Studies with a Jadad score of ≥3 were considered high quality, while those with a score of ≤2 were considered low quality. Disagreements were resolved through discussion with a third reviewer (ZY). In addition, we assessed risk of bias of each included study using the Cochrane Risk of Bias 2 (ROB 2) tool for randomized trials. The ROB 2 tool evaluates five domains: randomization process, deviations from intended interventions, missing outcome data, measurement of the outcome, and selection of the reported result. Each domain was judged as “low risk”, “some concerns”, or “high risk”, and an overall risk-of-bias judgement was assigned to each study. Two reviewers (LY and HZ) independently performed the assessment; disagreements were resolved by discussion with a third reviewer (ZY). Risk of bias was assessed at the outcome level using ROB2, overall study-level quality was evaluated with the Jadad scale.

### 2.7 GRADE assessment

We assessed the certainty of evidence for outcomes using the Grading of Recommendations Assessment, Development and Evaluation (GRADE) approach. The certainty was rated as high, moderate, low, or very low based on five domains: risk of bias, inconsistency, indirectness, imprecision, and publication bias. GRADH assessments were preformed using the GRADEpro GDT website (https://www.gradepro.org). Two reviewers (LY and HZ) independently rated the certainty of evidence, with disagreements resolved through discussion with a third reviewer (ZY).

### 2.8 Data synthesis

Statistical analyses were performed using STATA 12.0. For dichotomous outcomes (ADRs), we calculated the risk ratio (RR) with 95% confidence intervals (CIs) using a random-effects model. For continuous efficacy outcomes (MVV, incontinence episodes, micturition frequency), we initially attempted quantitative pooling using standardized mean differences (SMD). However, heterogeneity was substantial (all I^2^>58%), and with only three to five studies, subgroup analyses or meta-regression to explore sources of heterogeneity were not feasible. Consequently, we present the results narratively. For safety outcomes (ADRs), meta-analysis was performed as described above. Therefore, this systematic review included a meta-analysis only for safety outcomes, efficacy outcomes were presented narratively.

Studies were considered eligible for meta-analysis if they reported the same outcome with sufficient data to calculate effect sizes. For ADR meta-analyses, we extracted the number of events and total participants in each treatment group. If studies reported multiple doses of the same drug, we selected the dose that matched our primary intervention (solifenacin 5 mg) or comparator (solifenacin 10mg, mirabegron 50 mg). For combination therapy, we used data from the group receiving solifenacin 5 mg + mirabegron 50 mg.

We tabulated study characteristics in Table 1 and risk of bias assessments in Table 2. All meta-analyses were performed using STATA 12.0. Due to anticipated clinical and methodological diversity among the included studies, we used a random-effects model for all analyses to provide a more conservative estimate of the effect. Heterogeneity was assessed using the Cochrane Q test and quantified with the I^2^ statistic. An I^2^ value greater than 40% was considered indicative of substantial heterogeneity.

**TABLE 1:** Characteristics of included studies.

Studies [ref]	Country	Trials type	Sample size		Dosages	Duration of treatment	Outcomes
			Solifenacin	Control			
Gratzke et al. (2018)	Global	RCT	299	302	Solifenacin: 5 mg; mirabegron: 50 mg	12 months	Change from baseline to EOT in MVV per micturition, change from baseline to EOT in mean number of incontinence episodes, change from baseline to EOT in mean number of micturitions; ADRs
				1,193	Solifenacin: 5 mg; solifenacin + Mirabegron: 5 mg + 50 mg	12 months	Change from baseline to EOT in MVV per micturition, change from baseline to EOT in mean number of incontinence episodes, change from baseline to EOT in mean number of micturitions; ADRs
Mueller et al. (2019)	Global	RCT	303	305	Solifenacin: 5 mg; mirabegron: 50 mg	12 months	Change from baseline to EOT in mean number of incontinence episodes, change from baseline to EOT in mean number of micturitions; ADRs
				1,206	Solifenacin: 5 mg; solifenacin + Mirabegron: 5 mg + 50 mg	12 months	Change from baseline to EOT in mean number of incontinence episodes, change from baseline to EOT in mean number of micturitions; ADRs
Ozkidik et al. (2019)	Turkey	RCT	36	35	Solifenacin: 5 mg; mirabegron: 50 mg	12 months	Change from baseline to EOT in MVV per micturition, change from baseline to EOT in mean number of incontinence episodes, change from baseline to EOT in mean number of micturitions; ADRs
Haab et al. (2005)	Global	RCT	1,633	1,114	Solifenacin: 5 mg; solifenacin: 10 mg	52 weeks	Change from baseline to EOT in MVV per micturition, change from baseline to EOT in mean number of micturitions; ADRs
Staskin and Te (2006)	Global	RCT	578	1,233	Solifenacin: 5 mg; solifenacin: 10 mg	52 weeks	Change from baseline to EOT in mean number of incontinence episodes, change from baseline to EOT in mean number of micturitions; ADRs

RCT, randomized controlled trial; EOT, end of treatment; MVV, mean volume voided; ADRs, adverse drug reactions.

**TABLE 2:** Risk of bias of each studies included.

Studies	Description of randomization	Appropriate method for randomization	Description of double-blind	Appropriate method for double-blinding	Description of withdrawals and dropouts	Jadad scores	Quality
Gratzke et al. (2018)	1	1	1	1	1	5	High
Mueller et al. (2019)	1	1	1	1	1	5	High
Ozkidik et al. (2019)	1	1	0	0	1	3	High
Haab et al. (2005)	1	1	0	0	1	3	High
Staskin and Te (2006)	1	1	0	0	1	3	High

### 2.9 Subgroup analysis and investigation of heterogeneity

Pre-planned subgroup analyses were conducted based on the comparator drug (solifenacin 10mg, mirabegron 50mg, or combination therapy) to explore potential sources of heterogeneity in the outcomes. Meta-regression was planned but not performed due to the small number of included studies (<10 studies per outcome).

### 2.10 Sensitivity analysis

Sensitivity analyses were performed by sequentially omitting individual studies to assess the robustness of the pooled RRs for outcomes where substantial heterogeneity was present. Results of sensitivity analyses are presented in Supplementary Figures.

### 2.11 Publication bias

Publication bias for the primary outcomes was assessed qualitatively by visual inspection of a funnel plot and quantitatively using Begg’s and Egger’s tests, with P < 0.05 suggesting significant bias. For outcomes with fewer than 10 studies, funnel plot asymmetry should be interpreted with caution; therefore, we primarily relied on qualitative assessment for these outcomes.

## 3 Results

### 3.1 Study selection

**FIGURE 1:**
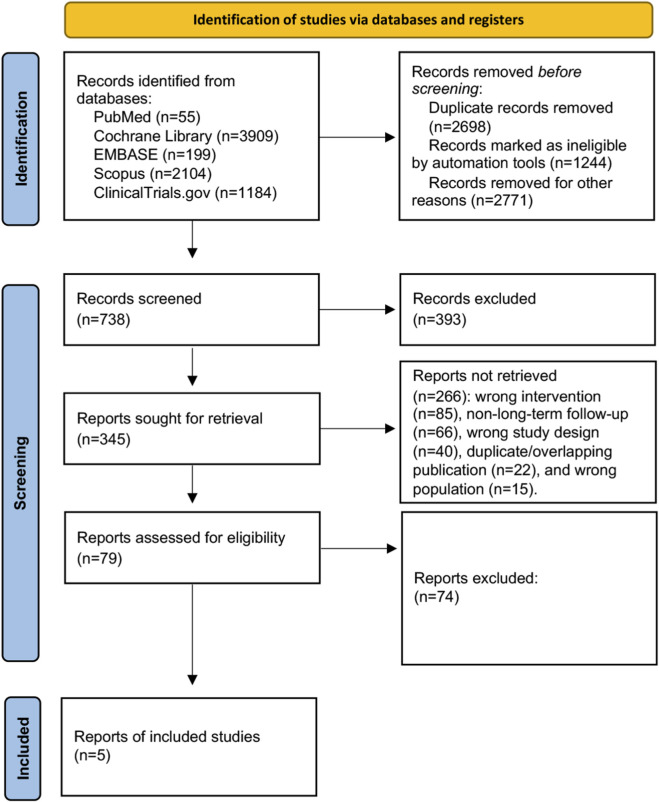
PRISMA 2020 flow diagram of study selection process.

The initial search yielded 7,451 records. After removing 2,698 duplicates, 738 records were screened based on titles and abstracts. Of these, 393 were excluded as they did not meet the eligibility criteria (e.g., wrong population, short-term treatment, irrelevant outcomes). The full texts of the remaining 345 articles were assessed for eligibility. A total of 266 full texts could not be obtained or were only available as conference abstracts. Among these, 38 studies met our PICOS criteria for a long-term solifenacin trial but lacked sufficient outcome data for inclusion. The remaining 228 full texts were excluded for other reasons: wrong intervention (n = 85, e.g., tolterodine, imidafenacin), non-long-term follow-up (n = 66), wrong study design (n = 40, e.g., review, case report, letter, observational study), duplicate/overlapping publication (n = 22), and wrong population (n = 15, e.g., paediatric, neurogenic bladder, non-OAB). A full-text review of 79 articles led to the exclusion of a further 74 studies for the following reasons: Four had unavailable full text after contacting authors; Seven were published protocols with no results; and 63 had no extractable data (e.g., duplicate publications, reviews, or studies reporting composite outcomes only). Ultimately, five RCTs (Gratzke et al., 2018; Mueller et al., 2019; Ozkidik et al., 2019; Haab et al., 2005; Staskin and Te, 2006) met all inclusion criteria and were included in this review (Figure 1).

### 3.2 Characteristics of the included studies

The five RCTs, published between 2005 and 2019, involved a total of 8,237 patients with OAB. All studies employed a parallel-group design and assessed long-term treatments (≥1 year). Interventions included solifenacin (5 mg or 10 mg), mirabegron 50mg, and combination therapy (solifenacin 5 mg + mirabegron 50 mg). The follow-up duration was 12 months in three studies, and 52 weeks in two studies. All studies used validated 7-day or 3-day electronic urinary diaries to assess efficacy outcomes. Detailed characteristics of the included studies are presented in Table 1 and Supplementary Table S3. Funding sources were reported in all five studies; four studies reported industry funding, and one study did not report funding sources.

### 3.3 Risk of bias of individual studies

All five included RCTs were assessed as high quality, with Jadad scores of three or higher (range 3–5). All studies described appropriate randomization methods; two studies described appropriate double-blinding procedures; and all studies adequately reported withdrawals and dropouts. Detailed Jadad scores for each study are provided in Table 2. The ROB 2 assessment results are summarized in Supplementary Figure S1. Overall, no study was judged to be at high risk of bias. The main issues identified were the inability to locate allocation concealment methods and the lack of trial registration information, which also raised concerns about selective reporting of results.

### 3.4 GRADE assessment

We assessed the certainty of evidence for the efficacy and safety outcomes using the GRADE approach (Supplementary Table S2). For the primary efficacy outcomes (incontinence episodes, micturition frequency, and MVV), the certainty of evidence was downgraded to low due to very serious imprecision (small number of studies, wide variance, and inability to compute a pooled estimate). For safety outcomes, certainty of evidence was rated as moderate for dizziness, sinusitis, urinary tract infection (UTI), nasopharyngitis, hypertension, vision blurred, and urinary retention due to imprecision (limited number of studies and relatively few events). However, the certainty of evidence for dry mouth and constipation was rated as moderate due to substantial statistical heterogeneity (I^2^>40%) across the included studies, warranting downgrading for inconsistency.

### 3.5 Study outcomes

Owing to high heterogeneity, efficacy outcomes were described narratively, pooled estimates were not presented.

#### 3.5.1 Change in the MVV per micturition from baseline to EOT

Three studies reported on MVV. Haab et al. found that long-term (52-week) treatment with solifenacin 5 mg significantly increased MVV from 147.6 mL to 187.4 mL, a 27% improvement (Haab et al., 2005). Gratzke et al. reported similar improvements in MVV with solifenacin 5 mg (Δ+25 mL) and mirabegron 50 mg (Δ+22 mL), but a significantly greater improvement with combination therapy (Δ+38 mL) compared to solifenacin monotherapy (P < 0.001) (Gratzke et al., 2018). Ozkidik et al. demonstrated comparable improvements in MVV with solifenacin 5 mg (Δ+36.04 mL) and mirabegron 50 mg (Δ+36.00 mL) (Ozkidik et al., 2019).

#### 3.5.2 Change in the mean number of incontinence episodes from baseline to EOT

All five studies reported on incontinence episodes. Haab et al. reported a reduction in mean 24-h incontinence episodes from 2.66 to 0.93 (Δ1.73) with solifenacin 5 mg (Haab et al., 2005). Staskin et al. found significant reductions with both solifenacin 5 mg (3.1–1.5) and 10 mg (3.2–1.3) compared to baseline (P < 0.05) (Staskin and Te, 2006). Mueller et al. showed that combination therapy was superior to monotherapies, particularly in patients aged≥65-year (Mueller et al., 2019). Ozkidik et al. reported comparable efficacy between solifenacin 5 mg and mirabegron 50 mg (Δ-1.9 episodes/3 days for both, P = 0.42) (Ozkidik et al., 2019). Gratzke et al. found that combination therapy (Δ-2.0) was superior to solifenacin 5 mg (Δ-1.9) and mirabegron 50 mg (Δ-1.6) (P < 0.05) (Gratzke et al., 2018).

#### 3.5.3 Change in the mean number of micturition events from baseline to EOT

All five reported on micturition frequency. Haab et al. found solifenacin 5 mg reduced mean 24-h micturitions from 12.16 to 9.18 (Δ-2.98) (Haab et al., 2005). Staskin et al. reported significant reductions with both solifenacin 5 mg (12.2–9.7) and 10 mg (11.7–9.1) (P < 0.001) (Staskin and Te, 2006). Mueller et al. found combination therapy was superior to monotherapies in patients <75 years (Mueller et al., 2019). Ozkidik et al. demonstrated comparable reductions with solifenacin 5 mg (Δ-0.08/3 days) and mirabegron 50 mg (Δ-0.11/3 days) (P = 0.37) (Ozkidik et al., 2019). Gratzke et al. confirmed the superiority of combination therapy (Δ-2.5) over solifenacin 5 mg (Δ-2.2) and mirabegon 50 mg (Δ-2.1) (P < 0.05) (Gratzke et al., 2018).

#### 3.5.4 Treatment-related ADRs: Dry mouth

**FIGURE 2:**
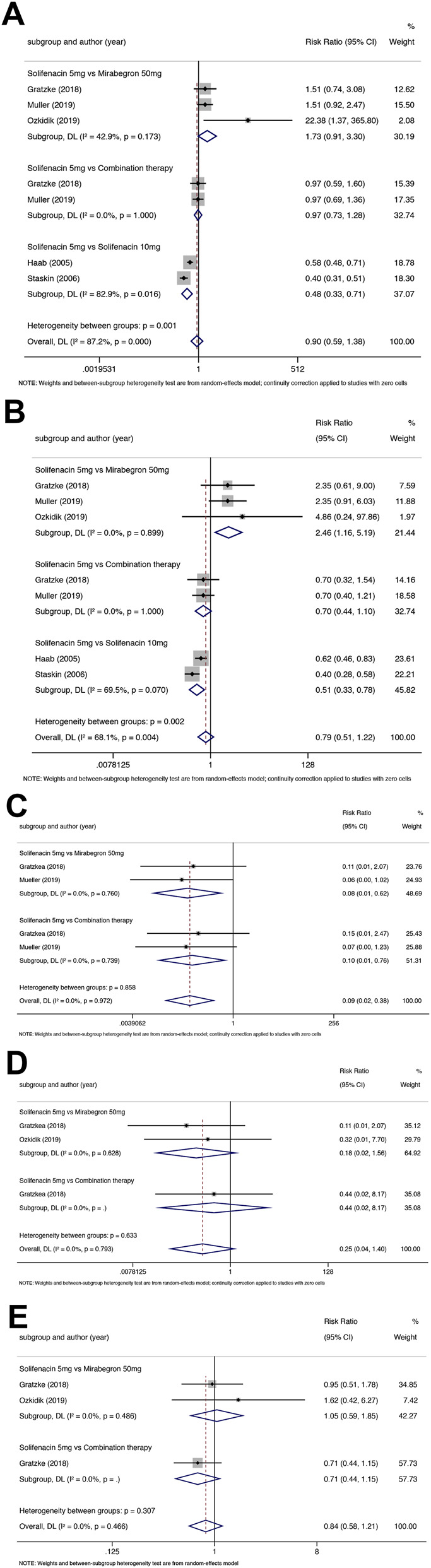
Forest plots of treatment-related adverse drug reactions (ADRs) comparing solifenacin 5 mg with control groups. **(A)** Dry mouth, **(B)** Constipation, **(C)** Dizziness, **(D)** Sinusitis, **(E)** Urinary tract infection. Risk ratios (RR) were calculated using a random-effects model. The size of each square is proportional to the study’s weight in the meta-analysis. Horizontal lines represent 95% confidence intervals (CIs). The diamond represents the pooled RR and its 95% CI for each subgroup.

Five studies (n = 8,257) reported on dry mouth (Figure 2A). Solifenacin 5 mg showed a non-significant trend toward a higher incidence of dry mouth compared to mirabegron 50 mg (RR 1.73, 95% CI 0.91 to 3.30; P = 0.10). No significant difference was observed compared to combination therapy (RR 0.97, 95% CI 0.73 to 1.28; P = 0.82). Solifenacin 5 mg was associated with a significantly lower risk of dry mouth compared to solifenacin 10 mg (RR 0.48, 95% CI 0.33 to 0.71; P < 0.001). Sensitivity analyses confirmed the robustness of these findings (Supplementary Figure S2).

#### 3.5.5 Treatment-related ADRs: Constipation

Five studies (n = 8,257) reported on constipation (Figure 2B). Solifenacin 5 mg was associated with a significantly higher risk of constipation compared to mirabegron 50 mg (RR 2.46, 95% CI 1.16–5.19; P = 0.02). No significant difference was found compared to combination therapy (RR 0.70, 95% CI 0.44–1.10; P = 0.12). Solifenacin 5 mg was associated with a significantly lower risk of constipation compared to solifenacin 10 mg (RR 0.51, 95% CI 0.33–0.78; P = 0.002). Sensitivity analyses confirmed the robustness of these findings (Supplementary Figure S3).

#### 3.5.6 Treatment-related ADRs: Dizziness

Two studies (n = 3,628) reported dizziness (Figure 2C). Solifenacin 5 mg was associated with a significantly lower risk of dizziness compared to both mirabegron 50 mg (RR 0.08, 95% CI 0.01–0.62; P = 0.02) and combination therapy (RR 0.10, 95% CI 0.01–0.76; P = 0.03).

#### 3.5.7 Treatment-related ADRs: Sinusitis

Two studies (n = 1,885) reported on sinusitis (Figure 2D). Solifenacin 5 mg showed a non-significant trend toward a lower incidence of sinusitis compared to mirabegron 50 mg (RR 0.18, 95% CI 0.02–1.56; P = 0.12). The risk was comparable between solifenacin 5 mg and combination therapy (RR 0.44, 95% CI 0.02–8.17; P = 0.58).

#### 3.5.8 Treatment-related ADRs: UTI

Two studies (n = 1,885) reported on UTI (Figure 2E). The incidence of UTI was comparable between solifenacin 5 mg and mirabegron 50 mg (RR 1.05, 95% CI 0.59–1.85; P = 0.88). Solifenacin 5 mg showed a non-significant trend toward a lower UTI incidence compared to combination therapy (RR 0.71, 95% CI 0.44–1.15; P = 0.17).

#### 3.5.9 Treatment-related ADRs: Nasopharyngitis, hypertension, vision blurred, urinary retention

Data for these ADRs are presented in the Supplementary Materials. The risk of nasopharyngitis (Supplementary Figure S4), hypertension (Supplementary Figure S5), blurred vision (Supplementary Figure S6), and urinary retention (Supplementary Figure S7) were generally comparable between solifenacin 5 mg and the other treatment groups.

#### 3.5.10 Patient-centered outcomes: Treatment persistence, quality of life, satisfaction, and cognitive effects

Four of the five included studies reported data on patient-centered outcomes beyond symptom diaries and adverse events.

##### 3.5.10.1 Treatment persistence/completion rates

Haab et al. reported that 81% (1,329/1,633) of patients completed the 40-week extension, and 91% of those who completed the double-blind trials opted to continue into the extension study. Gratzke et al. reported similar discontinuation rates across groups (combination 2.1%, solifenacin 1.7%, mirabegron 2.3% due to adverse events). Ozkidik et al. found that mean treatment duration was longer with mirabegron than with solifenacin (11.4 vs. 10.3 months, P = 0.042), and more patients discontinued solifenacin (13/17) primarily due to dry mouth.

##### 3.5.10.2 Quality of life (QoL)

Haab et al. reported that improvements in QoL (King’s Health Questionnaire) observed during double-blind treatment were maintained or further improved during long-term solifenacin therapy, with additional improvements of 28%–35% from the 12-week endpoint. Staskin et al. reported sustained improvements across all 10 domains of the King’s Health Questionnaire in patients with mixed urinary incontinence treated with solifenacin for up to 52 weeks, with the largest improvements in incontinence impact, role limitations, and physical limitations. Ozkidik et al. reported that Health-Related Quality of Life scores (HRQoL) improved similarly with solifenacin 5 mg (from 65.8 to 66.2) and mirabegron 50 mg (from 65.8 to 66.2) at 12 months, with no significant difference between groups.

##### 3.5.10.3 Patient satisfaction

Haab et al. reported that after 52 weeks of solifenacin, 85% of patients were satisfied with tolerability and 74% with efficacy. Staskin et al. found that 74% of patients rated solifenacin efficacy as “satisfactory” at the end of 52 weeks.

##### 3.5.10.4 Cognitive effects in older adults

Mueller et al., in a prespecified analysis of patients aged ≥65 and ≥75 years, explicitly noted that “there were no reports of confusion or cognitive changes during this study.” No other included studies systematically assessed or reported cognitive outcomes.

### 3.6 Publication bias

**FIGURE 3:**
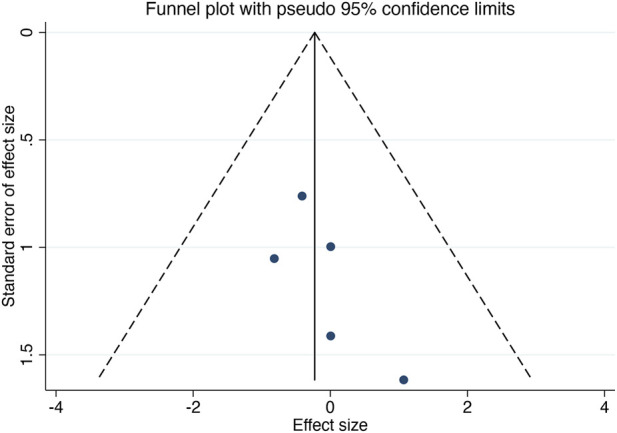
Funnel plot for publication bias assessment of the outcomes. The plot displays the log risk ratio against its standard error. Each circle represents an individual study comparison. The pseudo 95% confidence limits are shown as dashed lines. The symmetrical distribution suggests a low risk of publication bias.

Visual inspection of the funnel plot for the primary outcomes was symmetrical (Figure 3). Both Begg’s (P = 0.734) and Egger’s (P = 0.798) tests suggested no significant publication bias. However, these results should be interpreted with caution due to the small number of included studies.

## 4 Discussion

This systematic review and meta-analysis synthesized long-term (≥1 year) data from five high-quality RCTs to compare the efficacy and safety of solifenacin 5mg, mirabegron 50mg, and their combination for OAB. Our findings suggest that while combination therapy may offer superior efficacy across multiple outcomes, this benefit comes with a potential trade-off in tolerability for certain ADRs. In contrast, monotherapies, particularly mirabegron 50 mg, appear to balance efficacy with a more favorable ADR profile than combination therapy.

The narrative synthesis confirmed the superiority of combination therapy over each monotherapy for all efficacy outcomes. This aligns with the complementary mechanisms of action of the two drug classes and supports the clinical practice of considering combination therapy in patients with an inadequate response to monotherapy. Notably, as reported by Mueller et al. (Mueller et al., 2019), the superiority of combination therapy for incontinence was more pronounced in patients ≥65 years, while its benefit for reducing micturition frequency was greater in those <75 years, suggesting that patient age and symptom profile may influence the choice of combination therapy. The comparable efficacy observed between solifenacin 5 mg and mirabegron 50 mg monotherapies is consistent with previous short-term meta-analyses (He et al., 2023; Mostafaei et al., 2022; Luo et al., 2012).

The meta-analysis of ADRs revealed important differences in safety profiles, which are critical for long-term treatment adherence. Mirabegron 50 mg was associated with a significantly lower risk of constipation and a trend toward lower risk of dry mouth compared to solifenacin 5 mg. These findings are clinically significant as dry mouth and constipation are among the most common reasons for discontinuing antimuscarinic therapy. The higher risk of these ADRs with solifenacin is consistent with its anticholinergic mechanism of action. The dose-dependent relationship for solifenacin, with 10 mg carrying a higher risk of dry mouth and constipation than 5 mg, underscores the importance of using the lowest effective dose.

Conversely, solifenacin 5 mg was associated with a lower risk of dizziness compared to mirabegron 50 mg and combination therapy. While dizziness can affect quality of life, it is not typically cited as a primary driver of treatment discontinuation in OAB. The comparable risks for other ADRs, such as hypertension, UTIs, and nasopharyngitis, suggest these are less likely to be differentiating factors in drug choice.

Our narrative synthesis of patient-centered outcomes reinforces the clinical relevance of the ADR findings. Although persistence was not a primary endpoint in the included trials, the higher completion rates and longer treatment duration with mirabegron observed by Ozkidik et al., coupled with the high patient satisfaction reported for solifenacin (74%–85%) by Haab and Staskin, suggest that tolerability directly impacts real-world adherence. Importantly, the absence of cognitive adverse events in the elderly subgroup (Mueller et al.) provides some reassurance for long-term use of solifenacin and combination therapy in older patients, although systematic cognitive assessments were lacking in most studies. These patient-reported and functional outcomes are critical for long-term OAB management, as treatment success depends heavily on sustained adherence and perceived benefit.

Our findings are consistent with previous short-term studies and meta-analyses that have demonstrated comparable efficacy between solifenacin and mirabegron (He et al., 2023; Mostafaei et al., 2022; Luo et al., 2012). However, our study extends these findings by focusing on long-term (≥1 year) outcomes, which are more relevant for chronic OAB management. The superior efficacy of combination therapy observed in our study aligns with the 12-week SYNERGY study (Abrams et al., 2015), and our long-term data from SYNERGY II confirm that this benefit is sustained over 12 months.

Real-world evidence has suggested better persistence with mirabegron than with solifenacin (Kreydin et al., 2021; Mass-Lindenbaum et al., 2021), which may be explained by the lower incidence of bothersome anticholinergic ADRs such as dry mouth and constipation. Our findings provide a potential explanation for these real-world observations and support the use of mirabegron as a first-line option to improve treatment adherence.

A recent systematic review and meta-analysis compared mirabegron and solifenacin in children with OAB and found comparable efficacy with a more favorable safety profile for mirabegron (Shaheen et al., 2026). While this appears similar to our review, several key differences should be noted. First, the paediatric population is distinct from adults in terms of OAB aetiology, drug metabolism, and safety considerations, limiting direct comparability. Second, that review predominantly focused on short-term (12-week) outcomes, whereas our study specifically evaluated long-term (≥1 year) treatment. Third, the paediatric review did not include combination therapy (solifenacin + mirabegron), which we identified as more effective than either monotherapy. Therefore, our work complements rather than duplicates existing evidence, offering a focused evaluation of long-term pharmacological management in adults with OAB.

Our analysis synthesizes results primarily at the drug-dose level, yet the included studies exhibit substantial clinical and methodological heterogeneity that warrants careful consideration when interpreting the findings. Populations differ notably: Ozkidik 2019 is the only study that specifically enrolled post-surgical patients (100% female, median age 48 years), whereas Gratzke 2018, Mueller 2019, Haab 2005 and Staskin 2006 included non-surgical limited, mixed-gender cohorts with a median age of 56–63 years and approximately 78%–80% female participants. Therefore, the evidence from Ozkidik 2019 applies most directly to younger, female, post-surgical OAB patients, while the other studies better represent the general OAB population. Outcome measurement also varied: some studies used 7-day bladder diaries (higher reliability but lower adherence) and others used 3-day diaries (potentially greater intra-individual variability). Follow-up durations were similar (52 weeks vs. 12 months) and do not introduce meaningful temporal heterogeneity. These differences have direct implications for clinical interpretation and shared decision-making: for younger, female, post-surgical OAB patients, the results from Özkidik should guide practice; for older or mixed-gender patients, findings from Grätzke and Mueller provide more representative estimates. Clinicians should also recognize that absolute efficacy estimates may vary slightly depending on diary methodology, but relative treatment effects (e.g., combination therapy vs. monotherapy) remain robust across these methodological differences. Explicitly acknowledging these sources of heterogeneity allows for more nuanced, patient-centered application of our findings in routine practice.

This study has several limitations. First, the small number of included studies (n = 5) limits the generalizability and statistical power of our findings, particularly for less common ADRs. Second, substantial heterogeneity was present in some meta-analyses, which we explored through subgroup and sensitivity analyses, but which may reflect underlying differences in study populations or designs. Third, our analysis was limited to solifenacin and mirabegron due to a lack of long-term data for other agents in the same drug classes (e.g., tolterodine, vibegron). Therefore, our conclusions cannot be extended to all muscarinic receptor antagonists or β3-adrenoceptor agonists, and they apply solely to the specific drugs and doses studied.

Several limitations should be considered regarding potential bias. First, 38 studies that met our PICOS criteria for long-term solifenacin use were excluded because full-text results were unavailable (e.g., conference abstracts, trial protocols without results). Among these, sponsorship could not be determined for the majority, but the exclusion of unpublished studies may introduce publication and availability bias, potentially overestimating treatment effects. Second, while we used All-Fields searches in all databases, the possibility remains that combination trials where solifenacin is not mentioned anywhere in the indexed record could be missed. However, given that our search captured solifenacin regardless of field, this risk is low. Finally, most included studies were industry-sponsored. Given this substantial imbalance, formal sensitivity analysis by sponsorship was not feasible. We acknowledge that the predominance of industry-sponsored studies may introduce sponsorship bias.

Our review processes had several limitations. First, we were unable to perform quantitative synthesis for efficacy outcomes due to heterogeneous outcome reporting, precluding a pooled estimate of treatment effects. Second, we did not search for unpublished studies, which may have introduced publication bias. Third, while we extracted and narratively summarized available data on treatment persistence, quality of life, and patient satisfaction, these outcomes were not uniformly reported across studies, and no quantitative synthesis was possible. In particular, cognitive effects were only explicitly reported in one study (Mueller et al.), limiting conclusions for older adults. Future long-term trials should systematically collect and report these patient-centered outcomes to better inform shared decision-making. Additionally, this review did not assess treatment cost, comorbidity profiles, or detailed cognitive safety (beyond one study reporting no confusion events), which are important consideration in clinical decision-making as highlighted in the AUA/SUFU guideline framework. Additionally, while recent studies have explored mirabegron’s potential cardiovascular applications (Abdelhamid et al., 2026), our review did not systematically evaluate cardiovascular outcomes beyond the safety data reported in the included OAB trials. Future long-term studies should consider protocol-driven cardiovascular monitoring to better characterise the cardiac safety profile of mirabegron and combination therapy, particularly in older adults with pre-existing comorbidities.

Our findings have potential implications for the shared decision-making process recommended by the AUA/SUFU guidelines (Cameron et al., 2024), though they derive from a limited evidence base comprising only solifenacin 5 mg, mirabegron 50 mg, and their combination. For patients initiating pharmacological therapy with these specific regimens, our data suggest that mirabegron 50 mg may be considered a reasonable first-line choice. It offers comparable efficacy to solifenacin 5 mg in the studies reviewed, but with a lower risk of bothersome anticholinergic ADRs like constipation and dry mouth, which could potentially improve long-term persistence. However, for patients who are particularly concerned about dizziness, solifenacin might be discussed as an alternative. If a patient fails mirabegron monotherapy, our data support the option of switching to solifenacin monotherapy or adding it as combination therapy, with the understanding that combination therapy may increase efficacy but also the risk of certain ADRs like constipation and UTI. All these considerations should be balanced against patient comorbidities, cost, and cognitive risk, which were not analyzed in this review.

Future research should focus on: (1) long-term head-to-head comparisons of other OAB pharmacotherapies (e.g., tolterodine, vibegron, fesoterodine); (2) patient-reported outcomes and quality of life measures; and (3) identification of patient subgroups that may benefit most from specific treatments.

## 5 Conclusion

In this systematic review and meta-analysis of long-term (≥1 year) RCTs limited to solifenacin 5 mg, mirabegron 50 mg, and their combination, combination therapy was more effective than either monotherapy for key OAB symptoms, though with a potentially higher risk of certain ADRs. Mirabegron 50 mg monotherapy demonstrated a more favorable tolerability profile (lower risk of constipation and dry mouth) compared with solifenacin 5mg, while maintaining comparable efficacy. Among the specific regimens evaluated, our findings suggest that mirabegron 50 mg may be a reasonable first-line option for long-term management of OAB. However, this conclusion should be interpreted within the context of important limitations: the evidence base includes only one β3-adrenoceptor agonist (mirabegron) and one antimuscarinic (solifenacin). Long-term data for other agents (e.g., vibegron, tolterodine) are lacking, and we did not analyze outcomes such as treatment cost, comorbidity profiles, or cognitive risk in older adults. As recommended by the AUA/SUFU guidelines, treatment decisions should integrate patient values, tolerability, and these unexamined factors. Given the limited number of long-term studies available, further research is warranted to confirm these findings and to evaluate the long-term comparative effectiveness and safety of other OAB pharmacotherapies. Moreover, systematic collection of patient-centered outcomes such as treatment persistence, QoL, and cognitive effects should be prioritized in future trials to align with the chronic nature of OAB management.

## Supplementary material

SUPPLEMENTARY FIGURE S1 Risk of bias assessment using the Cochrane Risk of Bias 2 (ROB 2) tool.

SUPPLEMENTARY FIGURE S2 Sensitivity analysis of the incidence of dry mouth in the 5 mg solifenacin group and control group. Notes: CI, confidence intervals.

SUPPLEMENTARY FIGURE S3 Sensitivity analysis of the incidence of constipation in the 5 mg solifenacin group and control group. Notes: CI, confidence intervals.

SUPPLEMENTARY FIGURE S4 Outcome: Treatment-Related ADRs: Nasopharyngitis. Notes: ADRs, adverse drug reactions.

SUPPLEMENTARY FIGURE S5 Outcome: Treatment-Related ADRs: Hypertension. Notes: ADRs, adverse drug reactions.

SUPPLEMENTARY FIGURE S6 Outcome: Treatment-Related ADRs: Vision blurred. Notes: ADRs, adverse drug reactions.

SUPPLEMENTARY FIGURE S7 Outcome: Treatment-Related ADRs: Urinary retention. Notes: ADRs, adverse drug reactions.
