# Supplementary material for: Long-term efficacy and safety of solifenacin, mirabegron, and their combination for overactive bladder: a systematic review
Source: Front Pharmacol. 2026 Jun 19;17:1874436. doi: 10.3389/fphar.2026.1874436 (PMC13328022; doi:10.3389/fphar.2026.1874436)
Supplement: Supplementary file 1 [file Table1.DOCX]

**Supplementary materials**

**SUPPLEMENTARY TABLE 1 Searching strategy.**

| Database | Keywords | Hits |
| --- | --- | --- |
| PubMed | ("urinary bladder, overactive"[MeSH Terms] OR ("urinary"[All Fields] AND "bladder"[All Fields] AND "overactive"[All Fields]) OR "overactive urinary bladder"[All Fields] OR ("overactive"[All Fields] AND "bladder"[All Fields]) OR "overactive bladder"[All Fields] OR "OAB"[All Fields]) AND ("solifenacin succinate"[MeSH Terms] OR ("solifenacin"[All Fields] AND "succinate"[All Fields]) OR "solifenacin succinate"[All Fields] OR "solifenacin"[All Fields] OR "solifenacine"[All Fields]) AND "long-term"[All Fields] | 55 |
| Embase | (overactive AND ('bladder'/exp OR bladder) OR oab) AND ('solifenacin'/exp OR solifenacin) AND 'long term'  (searches all fields) | 199 |
| Cochrane Library | (overactive bladder) OR (OAB) AND (solifenacin) AND (long-term) (Cochrane indexes full text) | 3909 |
| Scopus | ( ALL ( overactive bladder ) OR ALL ( OAB ) AND ALL ( solifenacin ) AND ALL ( long-term ) ) (All searches full record) | 2104 |
| ClinicalTrials.gov | (overactive bladder) OR (OAB) AND (solifenacin) AND (long-term)  (searches all study fields) | 1184 |

Note: All searches were performed without restriction to title/abstract only. “All Fields” or database-equivalent full-text search was used.

**SUPPLEMENTARY TABLE 2 GRADE assessment for outcomes.**

| **Certainty assessment** | | | | | | | **№ of patients** | | **Effect** | | **Certainty** | **Importance** |
| --- | --- | --- | --- | --- | --- | --- | --- | --- | --- | --- | --- | --- |
| **№ of studies** | **Study design** | **Risk of bias** | **Inconsistency** | **Indirectness** | **Imprecision** | **Other considerations** | **solifenacin** | **other medicine** | **Relative (95% CI)** | **Absolute (95% CI)** |  |  |
| **Change in the MVV per micturition from baseline to EOT (follow-up: mean 12 months)** | | | | | | | | | | | | |
| 3 | randomised trials | not serious | not serious | not serious | Very serious^a^ | none | 1968 | 2644 | - | see comment | ⨁⨁◯◯ Low | CRITICAL |
| **Change in the mean number of incontinence episodes from baseline to EOT (follow-up: mean 12 months)** | | | | | | | | | | | | |
| 5 | randomised trials | not serious | not serious | not serious | Very serious^a^ | none | 2849 | 5388 | - | see comment | ⨁⨁◯◯ Low | CRITICAL |
| **Change in the mean number of micturition events from baseline to EOT** | | | | | | | | | | | | |
| 5 | randomised trials | not serious | not serious | not serious | Very serious^a^ | none | 2849 | 5388 | - | see comment | ⨁⨁◯◯ Low | CRITICAL |
| **Treatment-related ADRs: Dry mouth** | | | | | | | | | | | | |
| 5 | randomised trials | not serious | serious^a^ | not serious | not serious | none | 294/2849 (10.3%) | 792/5388 (14.7%) | **RR 0.90** (0.59 to 1.38) | **15 fewer per 1,000** (from 60 fewer to 56 more) | ⨁⨁⨁◯ Moderateb | CRITICAL |
| **Treatment-related ADRs: Constipation** | | | | | | | | | | | | |
| 5 | randomised trials | not serious | serious^a^ | not serious | not serious | none | 134/2849 (4.7%) | 382/5388 (7.1%) | **RR 0.79** (0.51 to 1.22) | **15 fewer per 1,000** (from 35 fewer to 16 more) | ⨁⨁⨁◯ Moderate^b^ | CRITICAL |
| **Treatment-related ADRs: Dizziness** | | | | | | | | | | | | |
| 2 | randomised trials | not serious | not serious | not serious | not serious | none | 0/606 (0.0%) | 51/3022 (1.7%) | **RR 0.09** (0.02 to 0.38) | **15 fewer per 1,000** (from 17 fewer to 10 fewer) | ⨁⨁⨁◯ Moderate^c^ | IMPORTANT |
| **Treatment-related ADRs: Sinusitis** | | | | | | | | | | | | |
| 2 | randomised trials | not serious | not serious | not serious | not serious | none | 0/339 (0.0%) | 9/1546 (0.6%) | **RR 0.25** (0.04 to 1.40) | **4 fewer per 1,000** (from 6 fewer to 2 more) | ⨁⨁⨁◯ Moderate^c^ | IMPORTANT |
| **Treatment-related ADRs: Urinary tract infection** | | | | | | | | | | | | |
| 2 | randomised trials | not serious | not serious | not serious | not serious | none | 23/339 (6.8%) | 123/1546 (8.0%) | **RR 0.84** (0.58 to 1.21) | **13 fewer per 1,000** (from 33 fewer to 17 more) | ⨁⨁⨁◯ Moderate^c^ | IMPORTANT |
| **Treatment-related ADRs: Nasopharyngitis** | | | | | | | | | | | | |
| 3 | randomised trials | not serious | not serious | not serious | not serious | none | 45/642 (7.0%) | 178/3057 (5.8%) | **RR 1.17** (0.92 to 1.51) | **10 more per 1,000** (from 5 fewer to 30 more) | ⨁⨁⨁◯ Moderate^c^ | IMPORTANT |
| **Treatment-Related ADRs: Hypertension** | | | | | | | | | | | | |
| 3 | randomised trials | not serious | not serious | not serious | not serious | none | 16/642 (2.5%) | 89/3057 (2.9%) | **RR 0.74** (0.47 to 1.14) | **8 fewer per 1,000** (from 15 fewer to 4 more) | ⨁⨁⨁◯ Moderate^c^ | IMPORTANT |
| **Treatment-Related ADRs: Vision blurred** | | | | | | | | | | | | |
| 3 | randomised trials | not serious | not serious | not serious | not serious | none | 91/2514 (3.6%) | 120/3858 (3.1%) | **RR 0.88** (0.67 to 1.16) | **4 fewer per 1,000** (from 10 fewer to 5 more) | ⨁⨁⨁◯ Moderate^c^ | IMPORTANT |
| **Treatment-Related ADRs: Urinary retention** | | | | | | | | | | | | |
| 3 | randomised trials | not serious | not serious | not serious | not serious | none | 4/642 (0.6%) | 24/3057 (0.8%) | **RR 0.80** (0.32 to 2.00) | **2 fewer per 1,000** (from 5 fewer to 8 more) | ⨁⨁⨁◯ Moderate^c^ | IMPORTANT |

Notes: CI: confidence interval; RR: risk ratio

#### Explanations

a. Downgrade two levels for the small number of trials, no pooled effect, and narrative synthesis.

b. Downgrade one level for the inconsistency because of the heterogeneity.

c. Downgrade one level for the small number of studies, low event counts, or wide confidence intervals for several comparisons.

**SUPPLEMENTARY TABLE 3 Characteristics of included studies: populations, interventions, and outcome definitions.**

| Study | %female | Median age | Surgery | Dosing pattern | Diary details | Duration |
| --- | --- | --- | --- | --- | --- | --- |
| Grztzke 2018 | 79.9% | 60 | Not limited | Solifenacin:5 mg; Mirabegron: 50 mg; Combination: 5 mg Solifenacin+50 mg Mirabegron | 7-day diary | 12 months |
| Mueller 2019 | 79.9% | 63 | Not limited | Solifenacin:5 mg; Mirabegron: 50 mg; Combination: 5 mg Solifenacin+50 mg Mirabegron | 7-day diary | 12 months |
| Ozkidik 2019 | 100% | 48 | Post-surgical | Solifenacin:5 mg; Mirabegron: 50 mg | 3-day diary | 12 months |
| Haab 2005 | 78% | 56 | Not limited | Solifenacin:5 mg; Solifenacin:10 mg | 3-day diary | 52 weeks |
| Staskin 2006 | 80% | Not mentioned | Not limited | Solifenacin:5 mg; Solifenacin:10 mg | 3-day diary | 52 weeks |


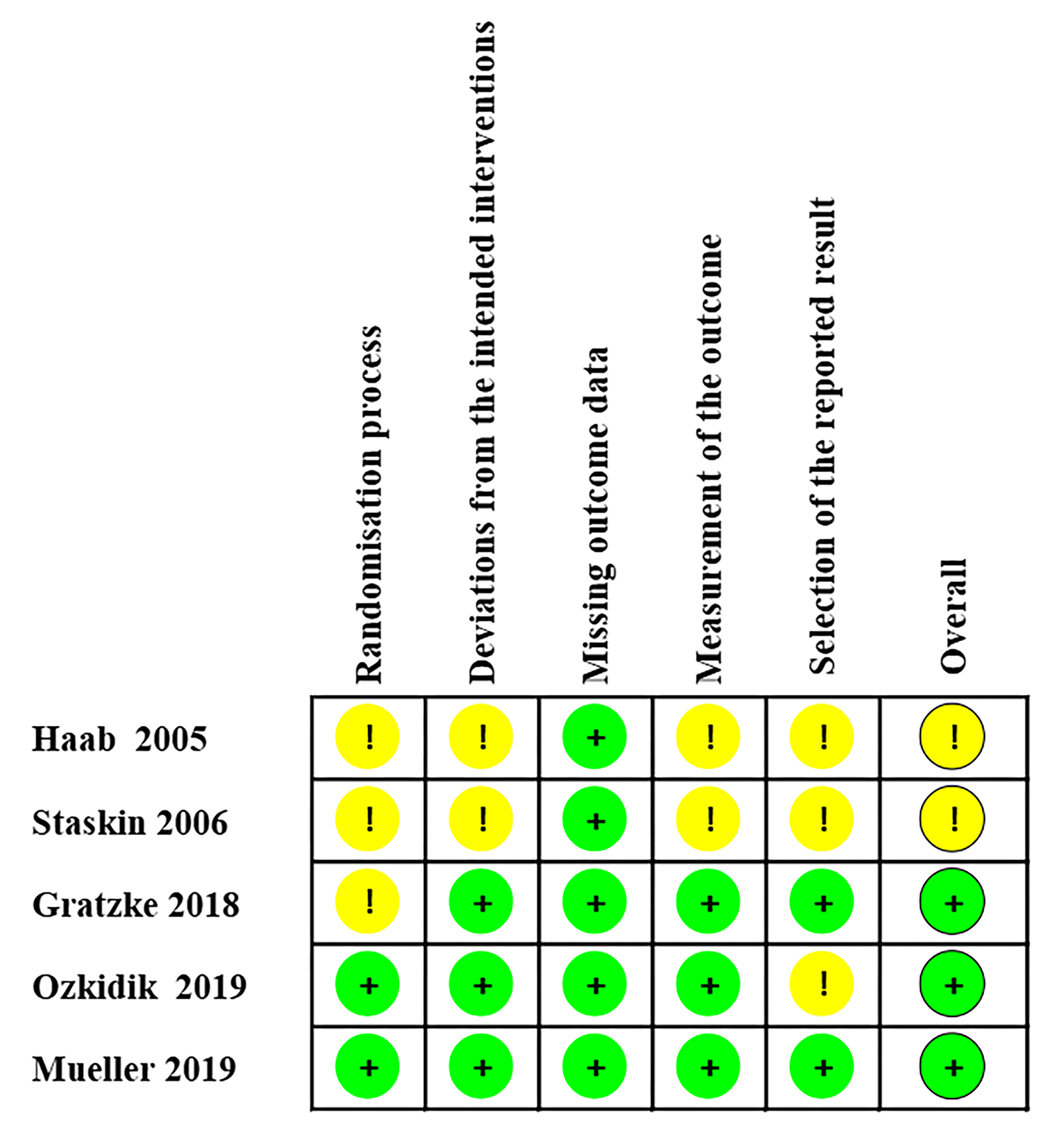


**SUPPLEMENTARY FIGURE 1**

Risk of bias assessment using the Cochrane Risk of Bias 2 (ROB 2) tool.

**
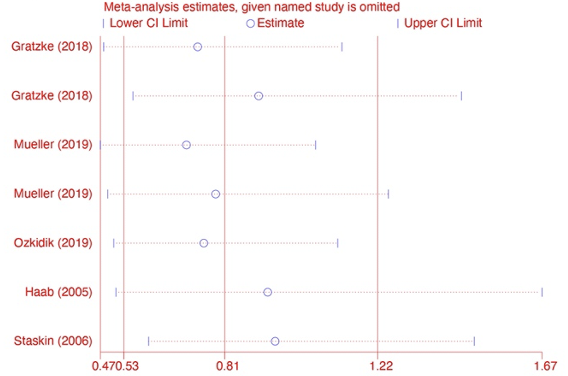
**

**SUPPLEMENTARY FIGURE 2**

Sensitivity analysis of the incidence of dry mouth in the 5mg solifenacin group and control group.

Notes: CI, confidence intervals.


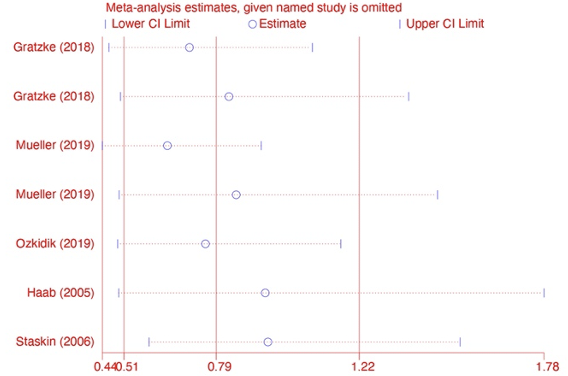


**SUPPLEMENTARY FIGURE 3**

Sensitivity analysis of the incidence of constipation in the 5mg solifenacin group and control group.

Notes: CI, confidence intervals.


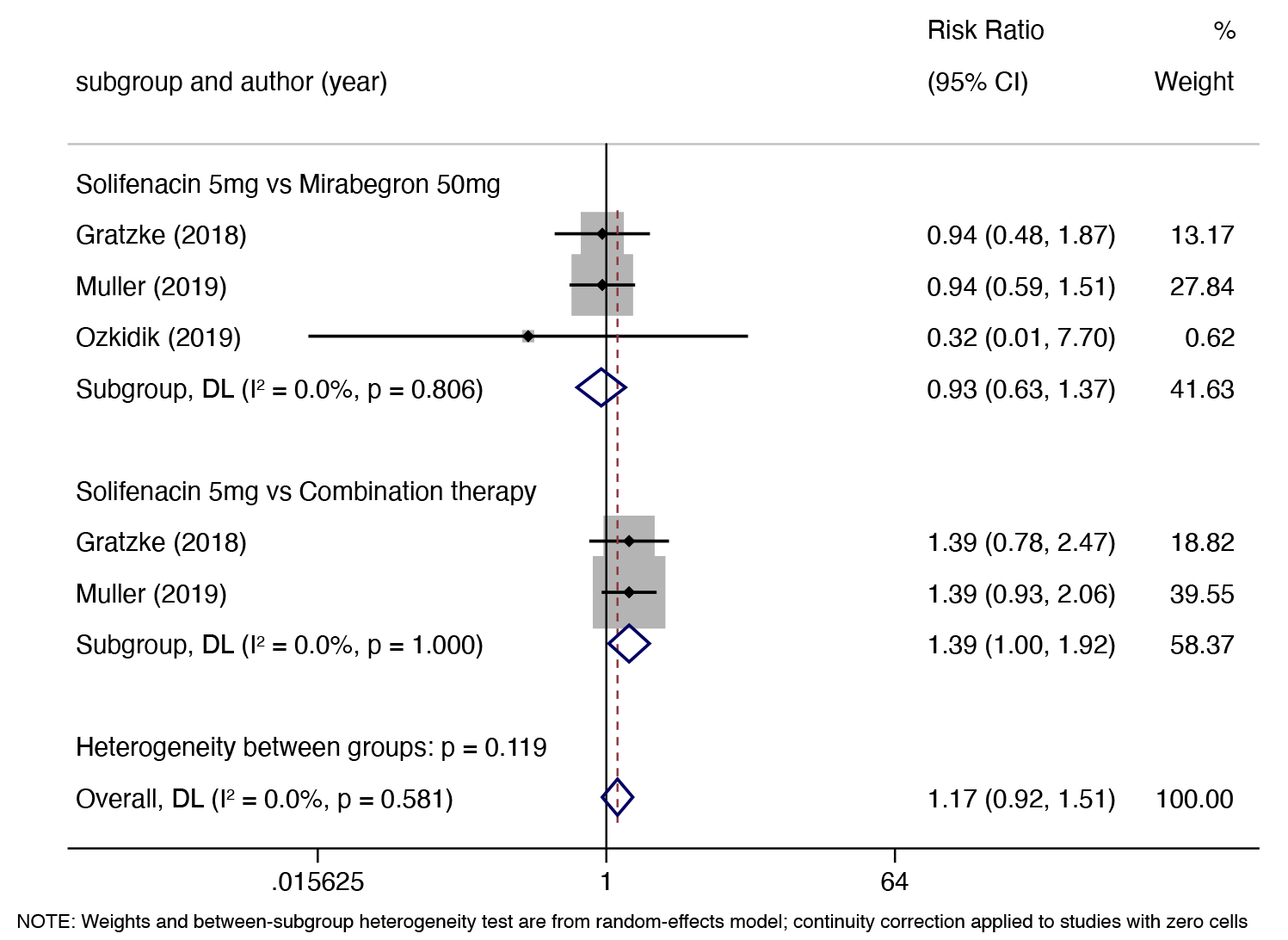


**SUPPLEMENTARY FIGURE 4**

Outcome: Treatment-Related ADRs: Nasopharyngitis.

Notes: ADRs, adverse drug reactions.


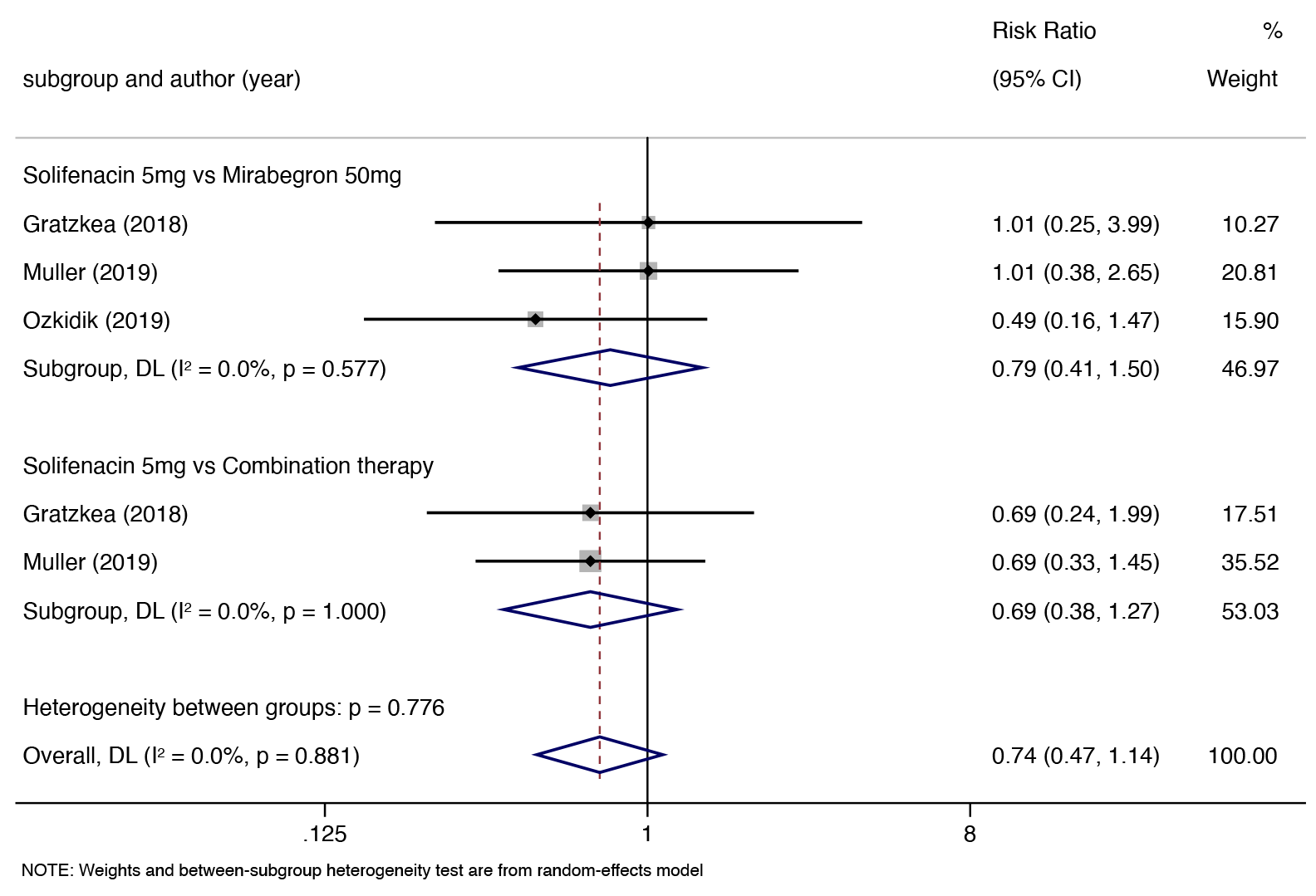


**SUPPLEMENTARY FIGURE 5**

Outcome: Treatment-Related ADRs: Hypertension.

Notes: ADRs, adverse drug reactions.


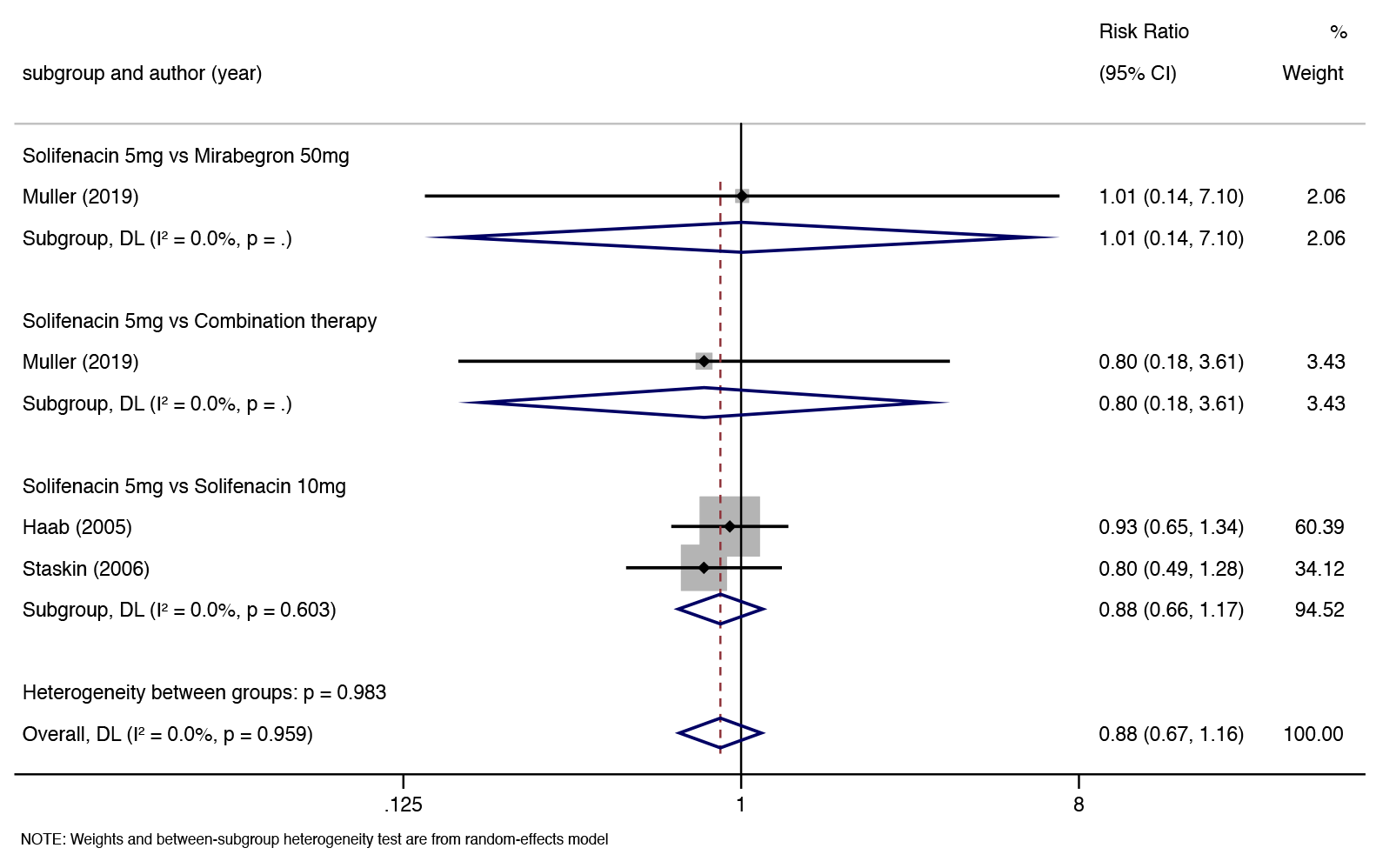


**SUPPLEMENTARY FIGURE 6**

Outcome: Treatment-Related ADRs: Vision blurred.

Notes: ADRs, adverse drug reactions.


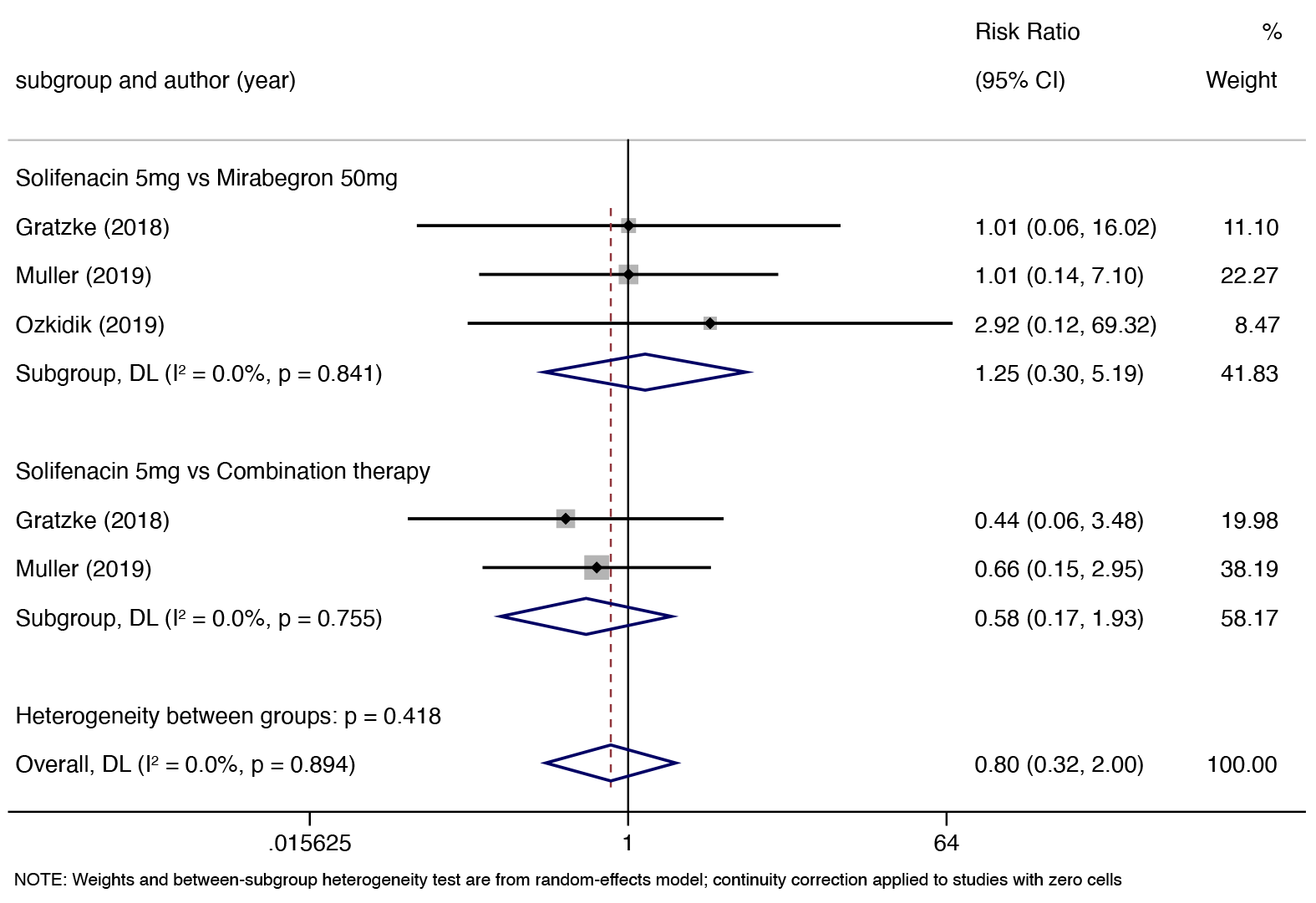


**SUPPLEMENTARY FIGURE 7**

Outcome: Treatment-Related ADRs: Urinary retention.

Notes: ADRs, adverse drug reactions.
